# Supportive data on the regulation of GLUT4 activity by 3-O-methyl-D-glucose

**DOI:** 10.1016/j.dib.2017.07.069

**Published:** 2017-07-28

**Authors:** Ofer Shamni, Guy Cohen, Arie Gruzman, Hilal Zaid, Amira Klip, Erol Cerasi, Shlomo Sasson

**Affiliations:** a The Institute for Drug Research, Section of Pharmacology, Diabetes Research Unit, Faculty of Medicine, The Hebrew University, Jerusalem 9112102, Israel; b Program in Cell Biology, Hospital for Sick Children, Toronto, OT, Canada M5G 1XB; c Endocrinology and Metabolism Service, Department of Internal Medicine, The Hebrew University-Hadassah Medical Center, Jerusalem 9112001, Israel

**Keywords:** Glucose transporter 4, Indinavir, Intrinsic activity, 3-O-methyl glucose, Myotubes

## Abstract

The data presented in this article are related to the research article entitled “Regulation of GLUT4 activity in myotubes by 3-O-methyl-D-glucose” (Shamni et al., 2017) [1]. These data show that the experimental procedures used to analyze the effects of 3-O-methyl-D-glucose (MeGlc) on the rate of hexose transport into myotubes were valid and controlled. The stimulatory effect of MeGlc was limited to glucose transporter 4 (GLUT4) and was independent of ambient glucose and protein synthesis. Cornish-Bowden kinetic analysis of uptake data revealed that MeGlc attenuated indinavir-induced inhibition of hexose transport in a competitive manner.

**Specifications Table**

**Table:** 

Subject area	Cell biology
More specific subject area	*Glucose transport regulation*
Type of data	*Text file, figures*
How data was acquired	*[^3^H]dGlc uptake assay and [^3^H]MeGlc transport assays*
Data format	*Analyzed*
Experimental factors	*Wild type- and GLUT4myc-expressing L6 cell were used. Primary cultures of bovine vascular endothelial and smooth muscle cell cultures were used as controls for GLUT1 expressing cells.*
Experimental features	*The uptake assay was performed usually in phosphate-buffered saline (PBS) buffer supplemented with 10 µM of deoxy-D-glucose (dGlc) and 37 kBq/mL of [^3^H]dGlc or 10 µM of MeGlc and 185 kBq/mL of [^3^H]MeGlc for 5 min at room temperature. The uptake was then terminated and the myotubes were lysed and taken for liquid scintillation counting. The results are given as pmol or nmol of dGlc or MeGlc, respectively, per mg protein, per min.*
Data source location	*Institute for Drug Research, The Hebrew University Faculty of Medicine, Jerusalem, Israel.*
Data accessibility	*The data are available with this article.*

**Value of the data**

• We have shown that MeGlc augments the intrinsic activity of GLUT4 in myotubes [1]. The data here show that the assays used to analyze the effect of the MeGlc on GLUT4 activity were valid and reproducible and that the experiments were well-controlled.
• The activity of GLUT1, in contrary to GLUT4, was not modified in the presence of MeGlc.
• The effect of MeGlc on GLUT4 was stereospecific.
• MeGlc reduced indinavir-induced inhibition of hexose transport by GLUT4 in a competitive manner.

## 1 Data

**Fig. 1:**
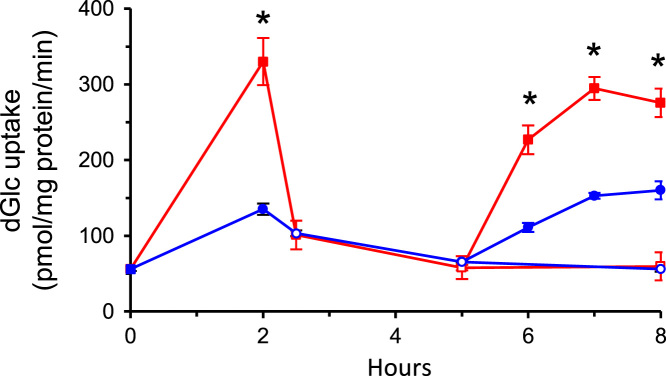
Repetitive MeGlc stimulations. L6 myotubes that had been maintained at 25 mM glucose for 24 h were washed and incubated in glucose-free α-Minimum Essential Medium (MEM) supplemented with 20 mM MeGlc (red squares) or 20 mM L-glucose (blue circles) for 2 h. The cultures were then washed and received glucose-free α-MEM supplemented with 25 mM glucose (open squares) or 20 mM L-glucose (L-Glc, open circles). The medium of some cultures was changed again 3 h later to glucose-free α-MEM supplemented with 20 mM MeGlc (red squares) or 20 mM L-Glc (blue circles), respectively. The standard [^3^H]dGlc uptake assay was performed at the indicated times. Results are mean±SEM, *n*=3, *P<0.05 in comparison with the respective L-glucose treatments.

**Fig. 2:**
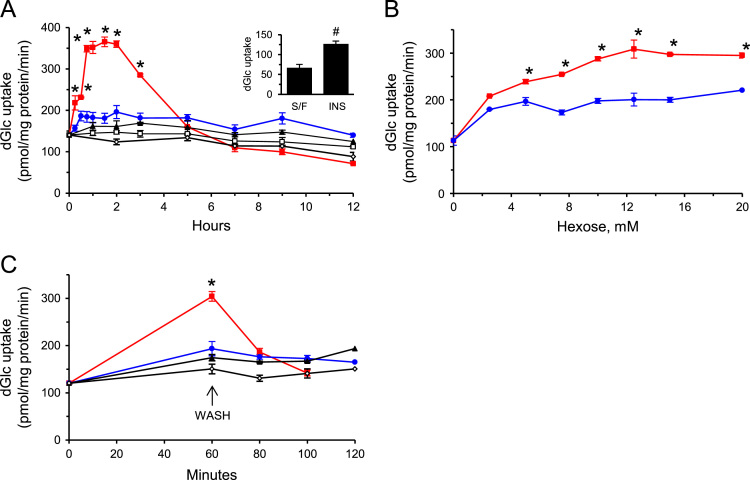
(A–C): MeGlc augments the rate of [^3^H]dGlc uptake in L6 myotubes at 5 mM glucose. *A*, *Time-course analysis*: Myotube cultures that had been maintained at 5 mM glucose for 24 h were washed with PBS and received glucose-free α-MEM (black triangles) or glucose-free α-MEM supplemented with 20 mM MeGlc (red squares) or with 20 mM L-glucose (blue circles). Control cells received α-MEM supplemented with the original glucose level (open black squares) or were maintained in the original culture medium (open black diamonds). All cultures were taken for the standard [3H]dGlc uptake assay. Results are mean±SEM, *n*=3. *P<0.05 in comparison with the respective L-glucose incubations. *Inset:* Insulin-stimulated hexose uptake as described [1]. B, Dose-response analysis: similar myotube cultures were incubated with glucose-free α-MEM supplemented with the indicated concentrations of MeGlc (red squares) or L-glucose (blue circles) and were further incubated for 1 h. All cultures were then taken for the standard [^3^H]dGlc uptake assay. Results are mean±SEM, *n*=3, *P<0.05 in comparison with the respective L-glucose treatments. *C, Off-rate of MeGlc effect:* Other cultures received glucose-free α-MEM (black triangles) or glucose-free α-MEM supplemented with 20 mM MeGlc (black squares) or 20 mM L-glucose (blue circles). Control cells (open black diamonds) were maintained in the original culture medium and were incubated for 1 h, washed and further incubated with α-MEM supplemented with 5 mM glucose. The standard [^3^H]dGlc uptake assay was performed at the indicated times. Results are mean±SEM, *n*=3. *P<0.05 in comparison with the respective L-glucose treatments.

**Fig. 3:**
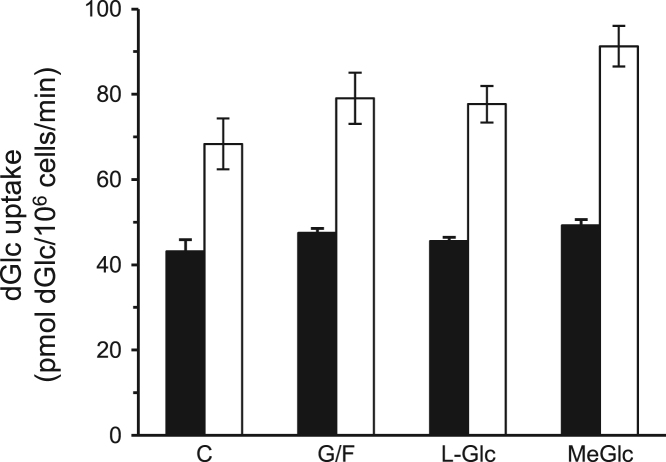
Lack of effect of MeGlc on hexose transport in vascular cells. Primary cultures of bovine aortic endothelial cells (black bars) and smooth muscle cells (white bars) were prepared as describe [2] and maintained with 5 mM glucose in the culture medium. These cultures were treated with glucose-free (G/F) medium, or 20 mM of L-Glucose (L-Glc) or MeGlc for 1 h and taken for the standard [^3^H]dGlc uptake assay. Results are mean±SEM, *n*=3.

**Fig. 4:**
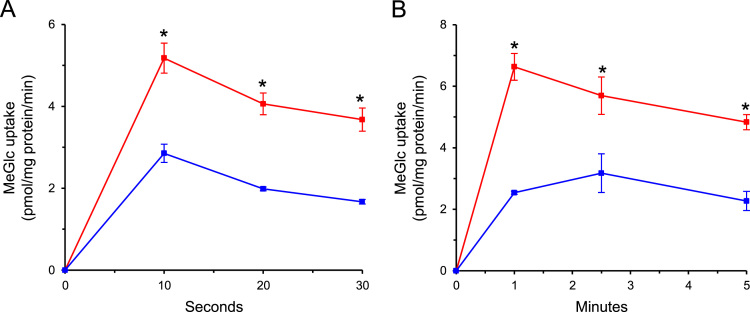
Time-course analysis of MeGlc transport. L6 myotubes were exposed to 20 mM MeGlc (red squares) or 20 mM L-glucose (blue circles) for 1 h and then were taken for the [^3^H]MeGlc transport assay at room temperature (left panel) or at 4 ºC (right panel) at the indicated times. Results are mean±SEM, *n*=3. *P<0.05 in comparison with the respective L-glucose treatment.

**Fig. 5:**
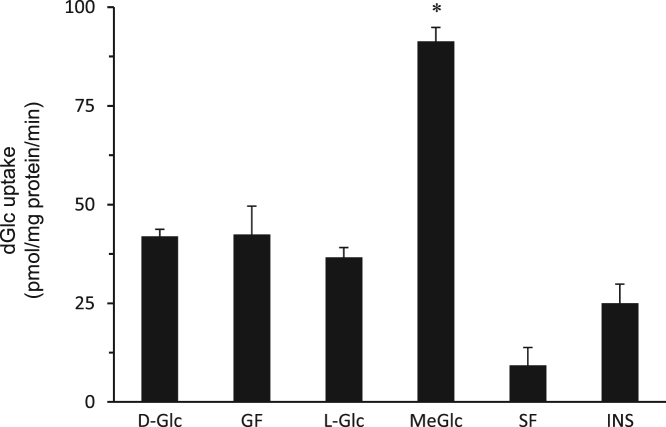
MeGlc augments the rate of [^3^H]dGlc uptake in L6-GLUT4*myc* myotubes. L6-GLUT4*myc* Myotube cultures that had been maintained at 25 mM glucose for 24 h were washed with PBS and received glucose-free α-MEM or glucose-free α-MEM supplemented with 20 mM MeGlc or with 20 mM L-glucose for 1 h. Control cells received α-MEM supplemented with the original glucose level (D-Glc, 25 mM). All cultures were taken for the standard [^3^H]dGlc uptake assay. Results are mean±SEM, *n*=3. *P<0.05 in comparison with the respective L-glucose incubations.

**Fig. 6:**
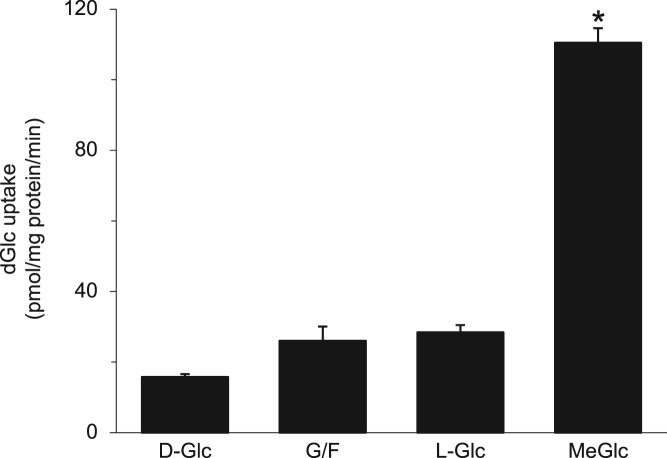
MeGlc stimulatory effect on dGlc uptake is preserved in L6- GLUT4*myc* myotubes under conditions resembling the conditions of the colorimetric assay. L6-GLUT4myc-expressing myotubes were treated as described in the legend to Fig. 5. The uptake assay was performed under the conditions described for the colorimetric assay, except for cell fixation, as specified under ‘Methods’ in [1]. Control cells (D-Glc) were left in the original culture medium. At the end of incubations, the cultures were washed three times with ice-cold PBS, placed on ice, and treated for 10 min with 5% (vol/vol) goat serum in ice-cold PBS supplemented with 20 mM L-glucose or 20 mM MeGlc, respectively. The buffer was then discarded and the cells were incubated with similar serum-free buffers for additional 60 min at 4 °C. The cells were then washed 5 times with ice-cold PBS, incubated again with the same buffers for additional 45 min, washed again 3 times and taken for a 15-min [^3^H]dGlc uptake assay at 4 °C. Results are mean±SEM, *n*=3. *P<0.05 in comparison with L-glucose treatment.

**Fig. 7:**
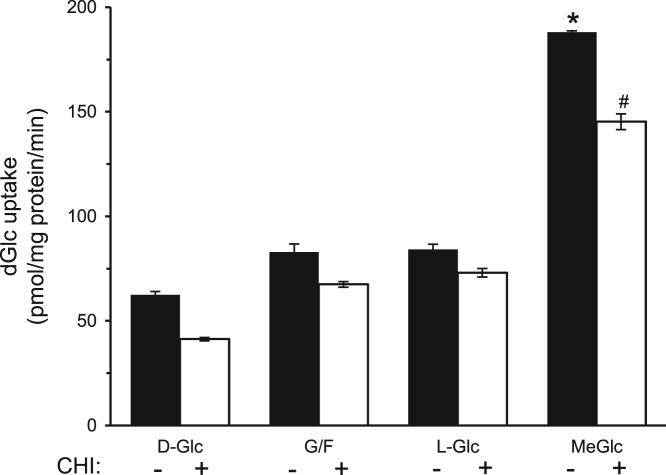
MeGlc augments hexose uptake in the presence of cycloheximide. L6 myotubes that had been maintained at 25 mM glucose for 24 h were exposed to 1 µM cycloheximide (CHI, white bars) or the vehicle (DMSO, black bars) during the last 4 h of incubation, then washed and further incubated for 1 h with α-MEM, supplemented with 25 mM glucose (D-Glc), 20 mM L-glucose (L-Glc) or 20 mM MeGlc, or with glucose-free (G/F) α-MEM, in the absence or presence of 1 µM cycloheximide. At the end of incubation the myotubes were washed three times and taken for the standard [^3^H]dGlc uptake assay. Results are mean±SEM, *n*=3. *P<0.05 in comparison with the respective L-glucose treatments.

**Fig. 8:**
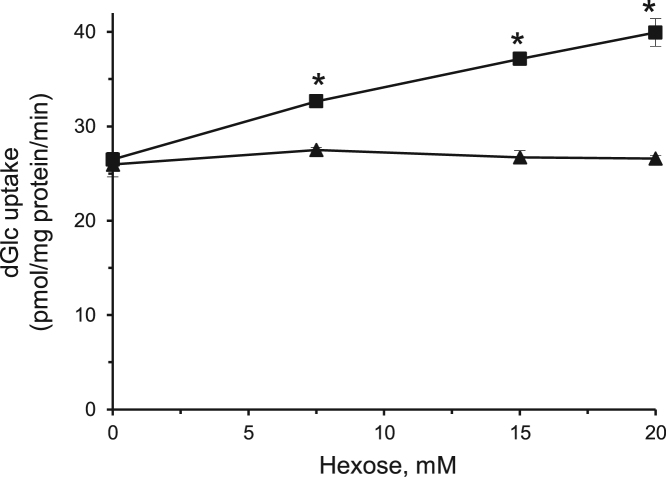
MeGlc stimulates hexose transport in the presence of glucose. L6 myotubes that had been maintained at 25 mM glucose for 24 h were supplemented with the indicated concentrations of MeGlc (squares) or L-Glc (triangles), incubated for an additional hour and taken for the standard [^3^H]dGlc uptake assay. Results are mean±SEM, *n*=3. *P<0.05 in comparison with the relative L-glucose treatment.

**Fig. 9:**
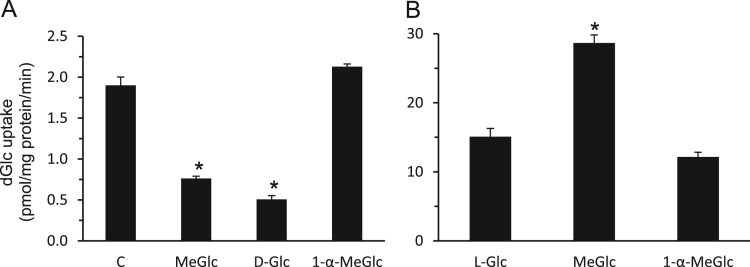
lack of effect of 1-α-Methyl-D-glucose on hexose transport in L6 myotubes. *A, Competition assay*: The rate of [^3^H]dGlc uptake was measured in L6 myotube cultures in the presence of 20 mM of non-radioactive Glc, MeGlc, L-Glc or 1-α-MeGlc in the uptake mixture*. B, Lack of effects of 1-α-MeGlc on hexose uptake.* Myotubes were treated as described and with 20 mM 1-α-MeGlc, L-Glc or MeGlc for 1 h. The cells were then washed and taken for the [^3^H]dGlc uptake assay. Results are mean±SEM, *n*=3. *P<0.05 in comparison with the respective L-Glc treatment.

**Fig. 10:**
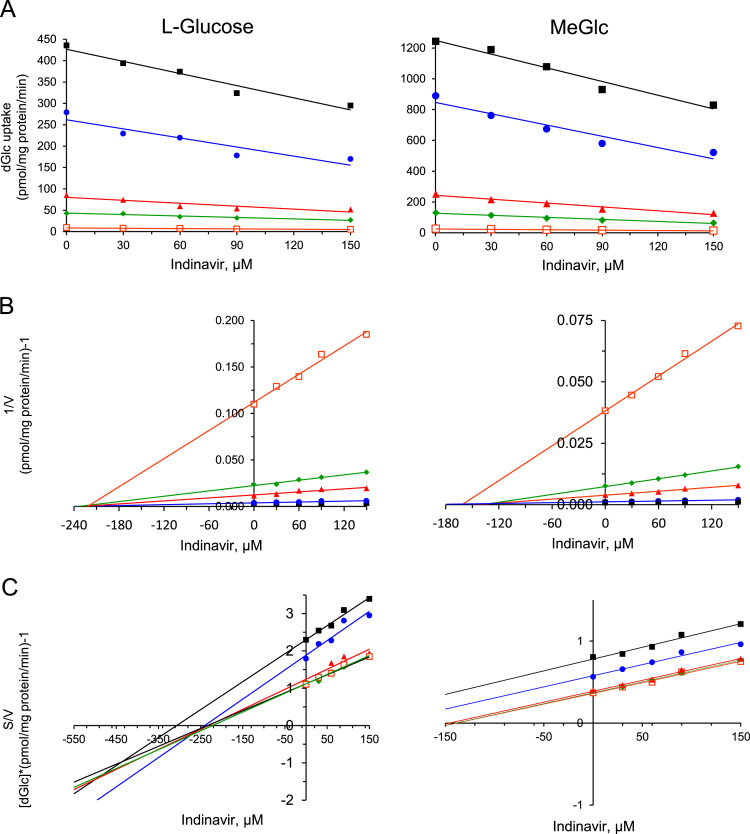
Kinetic analyses of indinavir-dependent inhibition of hexose uptake in L-Glc- and MeGlc-treated L6 myotubes. *A*, *Dose-response analysis of indinavir-induced inhibition of [*^*3*^*H]dGlc uptake in the presence of increasing concentrations of dGlc in the uptake assay:* L6 myotubes that had been maintained at 25 mM glucose for 24 h were incubated with 20 mM L-glucose (L-Glc) (left panel) or 20 mM MeGlc (right panel) for an additional hour. All cultures were then washed and taken for the standard [^3^H]dGlc uptake assay without or with the indicated indinavir concentrations in the presence of 10 μM (open orange squares), 50 μM (green diamonds),100 μM (red triangles), 0.5 mM (blue circles) or 1 mM (black squares) dGlc. *B, Dixon transformation*: The data obtained in *A* above were analyzed and plotted according to Dixon [3]*. C, Cornish-Bowden transformation*: The data obtained in *A* above were analyzed and plotted according to Cornish Bowden analysis [4].

The data presented here are supportive to the data presented in [1] with no duplications or overlap. The data in Fig. 1 show that repetitive exposure of L6 myotubes to MeGlc augmented the rate of hexose transport into L6 myotubes that were maintained at 25 mM glucose. Fig. 2 shows that MeGlc stimulated hexose uptake in L6 myotubes maintained at 5 mM glucose and that these effects were similar to those observed under 25 mM glucose (Fig. 2 in [1]). No such stimulatory effects of MeGlc were evident in GLUT1 expressing vascular cells (Fig. 3). Analyses of [^3^H]MeGlc transport into wild-type L6 myotubes (Fig. 4) and of [^3^H] dGlc uptake into L6 myotubes expressing GLUT4*myc* (Fig. 5, Fig. 6) confirm the validity and suitability of the assays used. Inhibition of protein synthesis with cycloheximide did not interfere with MeGlc effects (Fig. 7). MeGlc exerted its effects also in the presence of glucose in the uptake assay (Fig. 8). Unlike MeGlc, its analog 1-α-methylglucose (1-α-MeGlc) failed to modulate the hexose transport system (Fig. 9). Finally, Cornish-Bowden analysis of the uptake data shows that MeGlc attenuated indinavir-induced inhibition of hexose transport in a competitive manner (Fig. 10).

## 2 Experimental design, materials and methods

### 2.1 Cells and treatments

Wild-type L6 myotubes, L6 myotubes expressing GLUT4*myc* and primary cultures of bovine aortic endothelial and smooth muscle cells were treated as described and then taken for standard [^3^H]dGlc uptake or [^3^H]MeGlc transport assays.

## Funding information

The authors would like to acknowledge support from the Hebrew University Applied Research Fund A (2011), the Baby Seed Fund of the Yissum Research Development Company of the Hebrew University of Jerusalem (2011), and the Alex Grass Center for Drug Design and Synthesis at the Hebrew University (2013). O.S. and G.C. received fellowships from the Hebrew University Center for Diabetes Research.

## References

[1] Shamni O., Cohen G., Gruzman A., Zaid H., Klip A., Cerasi E., Sasson S. (2017 1859). Regulation of GLUT4 activity in myotubes by 3-O-methyl-D-glucose. Biochim. Biophys. Acta.

[2] Alpert E., Gruzman A., Totary H., Kaiser N., Reich R., Sasson S. (2002). A natural protective mechanism against hyperglycaemia in vascular endothelial and smooth-muscle cells: role of glucose and 12-hydroxyeicosatetraenoic acid. Biochem. J..

[3] Segel I.H. (1975). Enzyme Kinetics, Behavior and Analysis of Rapid Equilibrium and Steady-state Enzyme Systems.

[4] Cornish-Bowden A. (1974). A simple graphical method for determining the inhibition constants of mixed, uncompetitive and non-competitive inhibitors. Biochem. J..

